# Topography Prediction of Helical Transmembrane Proteins by a New Modification of the Sliding Window Method

**DOI:** 10.1155/2014/921218

**Published:** 2014-05-11

**Authors:** Maria N. Simakova, Nikolai N. Simakov

**Affiliations:** ^1^A.F. Mozhaisky Military Space Academy, Yaroslavl 150001, Russia; ^2^Yaroslavl State Technical University, Yaroslavl 150023, Russia

## Abstract

Protein functions are specified by its three-dimensional structure, which is usually obtained by X-ray crystallography. Due to difficulty of handling membrane proteins experimentally to date the structure has only been determined for a very limited part of membrane proteins (<4%). Nevertheless, investigation of structure and functions of membrane proteins is important for medicine and pharmacology and, therefore, is of significant interest. Methods of computer modeling based on the data on the primary protein structure or the symbolic amino acid sequence have become an actual alternative to the experimental method of X-ray crystallography for investigating the structure of membrane proteins. Here we presented the results of the study of 35 transmembrane proteins, mainly GPCRs, using the novel method of cascade averaging of hydrophobicity function within the limits of a sliding window. The proposed method allowed revealing 139 transmembrane domains out of 140 (or 99.3%) identified by other methods. Also 236 transmembrane domain boundary positions out of 280 (or 84%) were predicted correctly by the proposed method with deviation from the predictions made by other methods that does not exceed the detection error of this method.

## 1. Introduction

Problem and relevance of the study of membrane proteins, including GPCRs, are as follows. Membrane proteins are responsible for many cellular functions and processes, in particular ensuring the selective exchange of substances between the cell and its environment, maintaining the electric potential inside and outside the cell, and providing the transfer of electric signals into and out of the cell. They participate in nearly all energy transduction processes in the organism.

Protein functions are specified by its three-dimensional structure, which is usually obtained by X-ray crystallography [1, 2]. This method is directly applied to protein crystals, which must be produced beforehand using a very complex and laborious technique. The difficulty of handling membrane proteins during their production, purification, and crystallization due to protein instability, unfolding, aggregation, and heterogeneity has made it hard to solve their structures experimentally and to date the structure has only been determined for a very limited part of membrane proteins (<4%).

It is supposed that all information about the ultimate structure of a protein is contained in its amino acid sequence. Therefore, methods of computer modeling based on the data on the primary protein structure or the symbolic amino acid sequence have become an actual alternative to the experimental method of X-ray crystallography for studying the structure of membrane proteins [3].

From the variety of membrane proteins, the group of integral polytopic proteins (transmembrane proteins, TMPs) with multiple hydrophobic sites, domains permeating the membrane, is of considerable interest. Many of these proteins function as gateways or “loading docks” to transport specific substances and relay signals across the biological membrane.

The apparent feature and the inherent property of *α*-helical membrane proteins are the (possibly periodical) repetition of transmembrane domains consisting of hydrophobic amino acids (15–30 aa in length) [4]. If the mentioned repetition is periodic, it can be detected using the known method of Fourier transform, applied to a digital image of a symbolic sequence of amino acids in a protein, as was done in our previous works [4, 5].

If the repetition of transmembrane regions is aperiodic, it can be revealed by another method, that is, the method of the reiterated (four to five times) averaging of the protein hydrophobicity function in a window within the limits of 9–11 amino acids that moves along the sequence. This method is a novel advanced version of the known method of sliding window, which has been proposed and used in our previous work [4] to investigate the secondary structure of different membrane proteins.

The aim of the present work is to apply this method for the prediction of the characteristics of unknown secondary structures of TMPs, mainly of GPCRs; these characteristics specify the functional properties of the proteins.

G protein-coupled receptors (GPCRs), also known as seven-transmembrane domain receptors, comprise the largest family of membrane proteins in the human genome and the richest source of targets for the pharmaceutical industry [6].

Over 800 unique GPCRs have been revealed from human genome sequence analysis, approximately 460 of which are predicted to be olfactory receptors [7, 8]. The physiologic function of a large fraction of these 800 GPCRs is unknown. There are many obstacles to obtaining structures of GPCRs by X-ray crystallography; the major difficulties include poor protein stability and absence of homogeneity during crystallization due to inherent properties of these receptors [6, 9, 10].

Therefore, it is necessary to develop novel approaches in structurally resolving aspects of their biology [11–13]. One of such useful approaches is to screen these proteins with help of structural bioinformatics and methods of computer modeling to identify those of them with the best characteristics for structural studies and for crystallography trials.

## 2. Materials and Methods

We used the method of reiterated averaging hydrophobicity function within a sliding window over the amino acid sequence. Since TM domains (TMDs) consist predominantly of hydrophobic amino acids, it is evident that the average hydrophobicity for this region, as specified in the protein sequence by a function *f*(*k*) = *H*_*N*_[*i*(*k*)] of amino acid number *k* in the sequence, must be higher than that for both hydrophilic topological domains (TPDs) adjacent to it. Furthermore, this local property does not depend on the periodicity of the arrangement of characteristic TMDs and TPDs in the amino acid sequence. Here, *i*(*k*) = 1,2, …, 20 is the number of amino acids of the 20 known (Table 1), which is located at position *k* in the protein sequence.

For the first time, this idea was realized in [14], where averaging of the function *f*(*k*) within the limits of a segment, or window of width *d* = 5, 7, 9, 11, or 13 amino acids, moving along the amino acid sequence, was used. The result of averaging was assigned to a member of a new numerical sequence *f*_1_(*k*) with number *k* corresponding to the current position of the average segment point.

The scale of hydrophobicity *H*_*N*_(*i*) used in this method can be specified in different ways (Table 1) depending on the physically measured value that characterizes this property [14–20]. In [14–16], the change of value of free energy of amino acid side groups upon their transfer into water from a hydrophobic medium was used as a measure of hydrophobicity. In [17, 19], the measure (scale) of amino acid hydrophobicity was defined as the function *H*_4_(*i*) = 1 − 〈*A*〉/*A* ^0^ (Table 1) based on the values of the amino acid surface area *A* ^0^(*i*), which is available to solvent in the standard state, and the mean solvent accessible surface area 〈*A*(*i*)〉 in a folded protein conformation. In [17], the correlation between the free energy value and the surface area available to solvent was established.

The set of 20 amino acids can be divided into a few characteristic groups based on their degree of hydrophobicity by different ways. Thus, according to [19], we used the division of 20 amino acids into three groups by the degree of hydrophobicity, including hydrophobic (C, F, I, L, M, V, and W, seven in total), hydrophilic (D, E, G, K, N, P, Q, R, S, and T, ten in total), and neutral (A, H, and Y, three in total). The hydrophobic amino acids were assigned a value of +1, the hydrophilic amino acids were assigned a value of −1, and the neutral amino acids were assigned a value of 0. Thus, we obtained the crude scale *H*_3_(*i*) in Table 1. On another crude scale *H*_2_(*i*) the hydrophobic amino acids were assigned a value of +1, and the remaining amino acids were assigned a value of 0.

In our previous work [4], we proposed the procedure, different from that used in [14], for averaging the function *f*(*k*) on the scale *H*_*N*_(*i*). The averaging was carried out not once, but repeatedly, using the algorithm

(1) $$\begin{array}{c} f_{n}\left(k\right)=\frac{1}{2n+1}\overset{n}{\underset{k=-n}{\sum }}f_{n-1}\left(k\right),\;n=1,2,\ldots ,5,\\ f_{0}\left(k\right)=f\left(k\right), \end{array}$$

where every new averaging was performed on the previous function *f*_*n*−1_(*k*) over a window with a greater width *d* = 2*n* + 1; thus, the first averaging was over three elements, the second one was over five elements, and so on. In our opinion, the best result was obtained at *n* = 4 and the averaging over the window of width *d* = 9 amino acids (sometimes at *n* = 5 and *d* = 11 amino acids).

It is interesting to compare the values of the functions *f*_*n*_(*k*) with the characteristic value of the initial hydrophobicity function *f*_0_(*k*) = *f*(*k*), its arithmetic mean, calculated for the entire length *L* of the protein chain

(2) $$\begin{array}{c} u=\langle f\left(k\right)\rangle =\frac{1}{L}\overset{L}{\underset{k=1}{\sum }}f\left(k\right). \end{array}$$

For the major part of each hydrophobic region, in particular TMD, the correlation *f*_*n*_(*k*) > *u* must be performed, and in the hydrophilic region (TPD), a different correlation *f*_*n*_(*k*) < *u* must be performed.

The scale and function of hydrophobicity can be specified in different ways (there are more than 30 known ones). A comparison of different scales and functions of hydrophobicity carried out in our previous work [4] showed that the numbers and arrangements of transmembrane regions obtained upon their usage were often almost identical, even for very simple (rough) scales, for example, *H*_2_(*i*) and *H*_3_(*i*) (see Table 1). However, sometimes a particular scale can be preferable for a given protein due to the better resolution of closely spaced TMDs.

## 3. Results and Discussion

### 3.1. Testing of the Improved Method of a Sliding Window on Proteins with Known Structure

The improved method of a sliding window proposed in [4] by algorithm (1) was applied in this work to the group of membrane proteins, such as GPCRs, and to some other transmembrane *α*-helical proteins.

To further test the predictions of our method, first it was used to examine 5 proteins with already known structure (Table 2).

**Figure 1:**
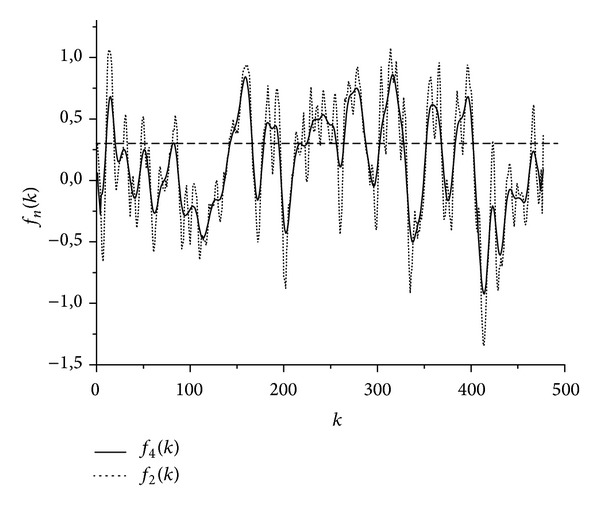
Hydrophobicity functions *f*_*n*_(*k*) for the protein P47871 in Table 2 after averaging at *n* = 2 and *n* = 4 on the scale *H*_5_(*i*) in Table 1; dotted line shows the level *u* = const = 0.266.

Figure 1 shows the results of averaging the hydrophobicity function for the protein sequence P47871 on the scale *H*_5_(*i*) in Table 1. Obviously, a hydrophobic segment in the form of a narrow peak relating to the signal peptide (SP) is present on the left edge of the graph of the function *f*_4_(*k*). If this peak is excluded, the remaining seven wide peaks that exceed the mean level *u* = const = 0.27 will just correspond to 7 TMDs in the resolved structure of this protein [21, 22]. In the graph of the function *f*_2_(*k*) the 2nd, the 3rd, the 5th, and the 7th TMDs have not been resolved yet, and there are several narrow peaks in their places.

**Figure 2:**
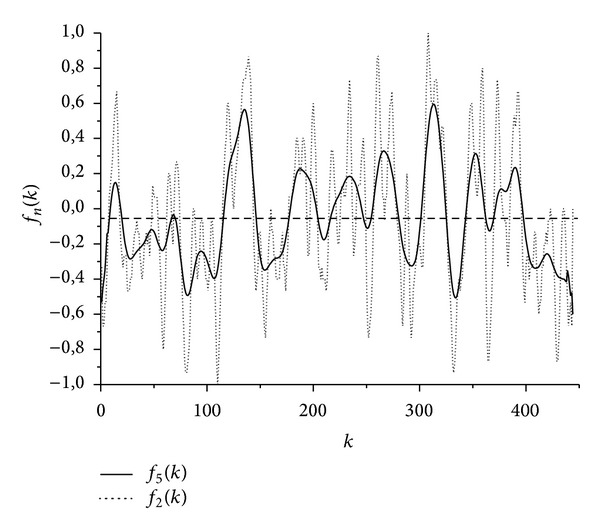
Hydrophobicity functions *f*_*n*_(*k*) for the protein P34998 in Table 2 after averaging at *n* = 2 and *n* = 5 on the scale *H*_3_(*i*) in Table 1; dotted line shows the level *u* = const = 〈*f*(*k*)〉 = −0.052.

Figure 2 shows the results obtained for the protein sequence P34998 using the relatively rough hydrophobicity scale *H*_3_(*i*) in Table 1. Apparently, a hydrophobic segment relating to the SP is revealed on the left edge of the graph of the function *f*_5_(*k*) above the mean level *u* = 〈*f*(*k*)〉 = −0.05, and also, in contrast to the function *f*_2_(*k*), all 7 TMDs known for the protein structure P34998 [21, 23] are resolved.

The boundaries of TMDs of different proteins were determined by the intersection of the graph of the function *f*_*n*_(*k*) with the straight line of some level *u* = const (e.g., the mean level *u* = 〈*f*(*k*)〉 for the whole protein sequence). They are summarized in Table 2 for 5 known proteins.

The TMD boundaries from [21] are also shown for comparison in Table 2.

Taking into account the errors Δ*k*_*b*_ ≈ *d*/2 ≈ 5 ⋯ 6 of the TMD boundary *k*_*b*_ detection, good agreement of the results of the TMD boundary position calculations with the data from [21] can be obtained. Indeed, according to Table 2, 34 TMDs out of 35 were resolved (or 97%); the obtained TMD boundary positions do not exceed the detection errors (Δ*k*_*b*_ ≤ 6) for 62 out of 70 boundaries (or 89%).

**Remark 1**

In the protein with a code P41595, the 2nd and the 3rd domains not resolved in calculating can be resolved using the outer boundaries of the combined segment of 89–151 aa by adding to the left border *k*_*b*_ = 89 and subtracting from the right border *k*_*b*_ = 151 the estimated average length of a domain 20 aa, as shown in Table 2 in a bold font.

In [21], a signal peptide (SP) consisting of 1–25 aa of a protein sequence is indicated in the structure of the protein P47871. In this part of the protein chain, the hydrophobic region of 11–23 aa was detected by the proposed method. Similarly, the sequence of the protein P34998 [21] contains a signal peptide consisting of 1–23 amino acid residues. The proposed method was helpful to reveal here the hydrophobic region of 9–19 aa.

It is worth noting that processing with reiterated (four to five times) averaging of the hydrophobicity function *f*_*n*_(*k*) on different scales (the rough scales *H*_2_(*i*) and *H*_3_(*i*) or the more precise scales *H*_4_(*i*)–*H*_7_(*i*)) produces different values for the TMD boundaries. Sometimes these differences are minor, but sometimes they are significant [4].

### 3.2. Comparison of Protein Secondary Structure Predictions Made by the Proposed Method and Other Techniques

Secondary structure predictions of a set of 20 membrane proteins belonging to a class of GPCRs performed using the new proposed method were compared with the predictions made by other methods (Table 3).

As can be seen from Table 3, the proposed method allowed revealing 139 TMDs out of 140 (or 99.3%) identified by other methods. In the protein P35414 (the last one in Table 3) the 6th and the 7th domains “merged” into one long stretch of 246–312 aa. However, taking into account Remark 1, the boundaries of these two domains can be easily recovered using the outer boundaries of the combined segment by adding to the left border *k*_*b*_ = 246 and subtracting from the right border *k*_*b*_ = 312 the estimated average length of a domain 20 aa, as shown in Table 3 in a bold font.

236 TMD boundary positions out of 280 (or 84%) were predicted correctly by the proposed method with deviation from the predictions made by other methods that does not exceed the detection error of this method (Δ*k*_*b*_ ≤ 6).

In [21], a signal peptide (SP) consisting of 1–21 aa of a protein sequence is indicated in the structure of the protein P25116. In this part of the protein chain the hydrophobic region of 6–17 aa was detected by the proposed method. Similarly, the sequence of the protein Q99835 [21] contains a signal peptide consisting of 1–27 amino acid residues. The proposed method was helpful to reveal here the hydrophobic region of 13–23 aa.

### 3.3. Predictions of Unknown Secondary Structure of GPCRs and Other Membrane Proteins

Then the proposed method of multiple averaging of hydrophobicity function was used to predict the location of hydrophobic regions, including TMDs, in several GPCRs with unknown structure. The results are shown in Table 4.

At least two hydrophobicity scales *H*_*N*_(*i*) were applied to make predictions for each of the 5 proteins. Obviously, these predictions are consistent with each other for most of the domain boundaries considering the detection errors Δ*k*_*b*_ = ±6.

For the protein B5D0C2 the calculation on the *H*_5_(*i*) scale resolved the 3rd and the 4th domains, but the application of the *H*_3_(*i*) scale did not resolve these domains; they merged into a single domain. And it was vice versa for the protein M9TID6 with the 6th and the 7th TMDs. Taking into account Remark 1, the boundaries of unresolved domains can be restored, as shown in Table 4 in a bold font.

Surprisingly, for the protein Q76L88 given that *f*_*n*_(*k*) is higher than the mean level *u* = 〈*f*(*k*)〉, only 6 domains were surely detected instead of 7 as for other proteins in Table 4.

The results of prediction of TMDs using the proposed method are shown in Table 5 for 4 *α*-helical membrane proteins of unknown structure. The first two proteins (P71044 and P49785) belong to the group of channels: intercellular, the third one Q8TMG0 to the group of methyltransferases, and the fourth one P77335 to the group of adventitious membrane proteins: alpha-helical pore-forming toxins.

Here, as well as in Table 4, the predictions were made on at least two hydrophobicity scales *H*_*N*_(*i*). Evidently, these predictions are consistent with each other for all domain boundaries considering the detection errors Δ*k*_*b*_ = ±6. Individual single domains predicted earlier by other methods [21] were also identified by the proposed method.

Table 6 shows data comparison from [21] with prediction of TMDs made by the proposed method for the long (*L* = 2424 aa) *α*-helical membrane protein from the group of adventitious membrane proteins: alpha-helical pore-forming toxins. Obviously, compliance between the predictions takes place for most of TMDs considering errors in determining their boundaries Δ*k*_*b*_ ≤ 6.

In the calculation using the proposed method of multiple averaging of hydrophobicity function over a sliding window, besides those domains indicated in Table 6, a hydrophobic region of 16–28 aa was identified, which may belong to a signal peptide (SP) or may be the 1st one out of 24 TMDs of the present protein. Moreover, it is obvious that TMDs numbered in [21] as 5, 11, 17, and 23 and highlighted in Table 6 by a bold font in our prediction have the numbers, which are one less than in [21], but other domains that are not specified in [21] have the numbers, which are one more. Thus, two varied predictions in Table 6 have great similarities as well as notable differences.

## 4. Conclusions

The first membrane protein topology prediction algorithms were based solely on the hydrophobicity plots, for example, [14, 16, 18], and it seemed that the performance of these early methods was rather poor in practice. Hence, they soon were supplied by novel statistical, machine-learning methods, which use hundreds of free parameters extracted from databases of experimentally mapped topologies [13, 27]. However, as it is stated in [27], the translocons (cellular machineries) responsible for membrane-protein biogenesis do not have access to statistical data but rather exploit molecular interactions to ensure that membrane proteins attain their correct topology. Therefore, as it is concluded in [13], those methods which are based on the same physical properties that determine translocon-mediated membrane insertion, by using properly scaled hydrophobicity values, may access the same level of prediction accuracy as the best statistical methods.

Thereby, here we presented the results of the study of 35 transmembrane proteins using cascade averaging of hydrophobicity function within the limits of a sliding window, as expressed in formula (1).

In the work [4], the proposed method was successfully applied to predict the location of TMDs, secondary structure elements of a number of membrane proteins, in particular, bacteriorhodopsin, halorhodopsin, sensory rhodopsin 2, some connexins, and others.

In the current work, this method was used to analyze the arrangement of the hydrophobic regions, including the transmembrane domains of another protein class, primarily GPCRs. At first, the method was tested on 5 known proteins of this class. Then an additional comparison of TMDs location predictions made by the proposed method and some other methods [21] was carried out on 20 proteins of the same class. These verifications confirmed the applicability of the proposed method for the stated purposes.

Whereupon, this method was used to predict the TMDs in proteins with unknown structure, namely, 5 GPCRs and 5 *α*-helical transmembrane proteins of other classes. For 9 out of 10 of these proteins (Tables 4 and 5) concordant predictions were made using at least two different hydrophobicity scales. The prediction made by the proposed method for a very long protein (Table 6) is consistent largely with the prediction made by another method [21].

These facts indicate the applicability and usefulness of the new method presented in our work [4] and proposed here.

## Figures and Tables

**Table 1:** Hydrophobicity scales *H*_*N*_(*i*).

*i*	Code	Abbreviation	Name	*H*_1_(*i*), [14]	*H*_2_(*i*), [19]	*H*_3_(*i*), [19]	*H*_4_(*i*), [17, 19]	*H*_5_(*i*), [18]	*H*_6_(*i*), [16]	*H*_7_(*i*) [20]
1	A	Ala	Alanine	1.8	0	0	0.74	0.62	1.60	−0.17
2	C	Cys	Cysteine	2.5	1	1	0.91	0.29	2.00	0.24
3	D	Asp	Aspartic acid	−3.5	0	−1	0.62	−0.90	−9.20	−1.23
4	E	Glu	Glutamic acid	−3.5	0	−1	0.62	−0.74	−8.20	−2.02
5	F	Phe	Phenylalanine	2.8	1	1	0.88	1.19	3.70	1.13
6	G	Gly	Glycine	−0.4	0	−1	0.72	0.48	1.00	−0.01
7	H	His	Histidine	−3.2	0	0	0.78	−0.40	−3.00	−0.96
8	I	Ile	Isoleucine	4.5	1	1	0.88	1.38	3.10	0.31
9	K	Lys	Lysine	−3.9	0	−1	0.52	−1.50	−8.80	−0.99
10	L	Leu	Leucine	3.8	1	1	0.85	1.06	2.80	0.56
11	M	Met	Methionine	1.9	1	1	0.85	0.64	3.40	0.23
12	N	Asp	Asparagine	−3.5	0	−1	0.63	−0.78	−4.80	−1.23
13	P	Pro	Proline	−1.6	0	−1	0.64	0.12	−0.20	−0.45
14	Q	Gln	Glutamine	−3.5	0	−1	0.62	−0.85	−4.10	−0.58
15	R	Arg	Arginine	−4.5	0	−1	0.64	−2.53	−12.3	−0.81
16	S	Ser	Serine	−0.8	0	−1	0.66	−0.18	0.60	−0.13
17	T	Thr	Threonine	−0.7	0	−1	0.70	−0.05	1.20	−0.14
18	V	Val	Valine	4.2	1	1	0.86	1.08	2.60	−0.07
19	W	Trp	Tryptophan	−0.9	1	1	0.85	0.81	1.90	1.85
20	Y	Tyr	Tyrosine	−1.3	0	0	0.76	0.26	−0.70	0.94

**Table 2:** Comparison of TMD boundaries calculated upon processing of hydrophobicity functions *f*_*n*_(*k*) at *n* = 3, 4, 5 on *H*_*N*_(*i*) (*N* = 3 and 5) scales for GPCRs with known data from [21].

Protein name, code, length	Data source	Number and boundaries of transmembrane domains						
	Scale level	1	2	3	4	5	6	7
GLR_ HUMAN P47871 477 aa	[21, 22]	137–161	174–198	226–249	264–285	304–326	351–369	382–402
	*H*_5_(*i*), *n* = 4 *u* = 0.266	143–166	180–192	218–257	261–288	303–327	353–368	384–401

CRFR1_ HUMAN P34998 444 aa	[21, 23]	112–142	179–203	219–247	255–282	299–324	336–360	368–397
	*H*_3_(*i*), *n* = 5 〈*u*〉 = −0.052	116–146	178–204	217–247	255–280	302–325	344–362	370–397

ADRB1_ MELGA P07700 483 aa	[21, 24]	39–67	77–103	116–137	156–179	206–231	286–315	321–343
	*H*_3_(*i*), *n* = 4, *u* = 0.1	44–64	81–99	108–138	160–181	214–229	293–314	320–331

5HT1B_ HUMAN P28222 390 aa	[21, 25]	50–75	85–110	124–145	166–187	206–228	316–336	350–371
	*H*_5_(*i*), *n* = 4 *u* = 0.243	46–72	86–109	119–145	168–185	205–230	316–340	343–369
	*H*_5_(*i*), *n* = 3 〈*u*〉 = 0.182	45–73	85–110	118–145	168–185	205–229	316–340	344–370

5HT2B_ HUMAN P41595 481 aa	[21, 26]	57–79	91–113	130–151	172–192	217–239	325–345	361–382
	*H*_5_(*i*), *n* = 5 〈*u*〉 = 0.164	54–81	89– **89–109**	−151 **131–151**	173–194	215–243	325–352	356–381

**Table 3:** Comparison of TMD boundaries calculated upon processing of hydrophobicity functions *f*_*n*_(*k*) at *n* = 4, 5 on *H*_*N*_(*i*) (*N* = 3, 5, 6) scales for GPCRs with known data from [21].

Protein name, code, length	Data source	Number and boundaries of transmembrane domains						
	Scale level	1	2	3	4	5	6	7
S1PR1_ HUMAN P21453 382 aa	[21], by similarity	47–71	79–107	122–140	160–185	202–222	256–277	294–314
	*H*_5_(*i*), *n* = 5 *u* = 0.25	48–69	83–107	122–142	160–195	199–223	255–281	293–310

ACM2_ HUMAN P08172 466 aa	[21], by similarity	23–45	60–80	98–119	140–162	185–207	389–409	424–443
	*H*_3_(*i*), *n* = 5 *u* = 0.07	21–48	60–85	90–122	142–167	192–208	389–415	422–429

ACM3_ RAT P08483 589 aa	[21], by similarity	67–90	104–124	142–163	184–206	229–251	492–512	527–546
	*H*_5_(*i*), *n* = 5 *u* = 0.30	62–92	105–128	137–161	187–208	221–249	492–515	526–541

CXCR1_ HUMAN P25024 350 aa	[21], potential	40–66	76–96	112–133	155–174	200–220	243–264	286–308
	*H*_3_(*i*), *n* = 4 *u* = −0.05	39–67	76–96	102–141	152–175	199–230	241–267	291–308

CCR5_ HUMAN P51681 352 aa	[21], potential	31–58	69–89	103–124	142–166	199–218	236–260	278–301
	*H*_5_(*i*), *n* = 5 *u* = 0.25	33–56	68–93	100–136	141–164	196–218	238–264	288–299

HRH1_ HUMAN P35367 487 aa	[21], potential	30–49	64–83	102–123	146–165	190–210	419–438	451–470
	*H*_5_(*i*), *n* = 5 *u* = 0.17	25–50	63–93	96–122	147–167	188–212	418–442	449–469

OPRK_ HUMAN P41145 380 aa	[21], potential	59–85	96–117	133–154	174–196	223–247	276–299	312–333
	*H*_6_(*i*), *n* = 4 *u* = 0.50	56–83	99–122	143–151	180–195	227–248	277–300	302–320

OPRM_ MOUSE P42866 398 aa	[21], potential	65–94	104–121	144–163	194–209	235–257	281–303	312–328
	*H*_3_(*i*), *n* = 4 *u* = −0.02	68–95	105–114	136–162	187–205	229–262	280–306	317–325

OPRD_ MOUSE P32300 372 aa	[21], potential	46–75	85–102	125–144	175–190	216–238	262–284	294–310
	*H*_5_(*i*), *n* = 5 *u* = 0.213	44–74	85–102	112–142	167–187	211–236	263–286	296–319

OPRX_ HUMAN P41146 370 aa	[21], potential	51–77	88–109	125–146	166–188	212–236	265–288	301–322
	*H*_3_(*i*), *n* = 5 *u* = 0.011	42–79	90–107	112–130	172–186	212–241	263–284	301–335

NTR1_ RAT P20789 424 aa	[21], potential	65–87	97–121	144–165	189–210	236–260	309–330	349–372
	*H*_5_(*i*), *n* = 5 *u* = 0.144	63–86	103–139	154–172	191–208	220–268	306–324	338–374

PAR1_ HUMAN P25116 425 aa	[21], potential	103–128	138–157	177–198	219–239	269–288	312–334	351–374
	*H*_3_(*i*), *n* = 4 *u* = 0.100	101–133	136–158	175–208	221–238	270–296	313–338	350–371

O51E1_ HUMAN Q8TCB6 317 aa	[21], potential	28–48	57–77	102–122	142–162	199–219	239–259	275–295
	*H*_5_(*i*), *n* = 5 〈*u*〉 = 0.300	12–49	60–77	80–120	146–166	198–227	243–260	276–292

SMO_ HUMAN Q99835 787 aa	[21], potential	234–254	263–283	315–335	359–379	403–423	452–472	525–545
	*H*_3_(*i*), *n* = 5 *u* = 0.00	236–251	264–283	313–340	362–380	403–425	451–473	519–545

GP160_ HUMAN Q9UJ42 338 aa	[21], potential	24–44	59–79	94–114	137–157	178–198	245–265	269–289
	*H*_5_(*i*), *n* = 5 *u* = 0.420	26–40	59–81	97–118	139–157	182–202	244–271	274–292

HRH3_ HUMAN Q9Y5N1 445 aa	[21], potential	40–60	71–91	109–129	157–177	197–217	360–380	396–416
	*H*_3_(*i*), *n* = 5 *u* = 0.00	33–61	72–95	105–132	155–173	191–222	360–388	395–416

HRH4_ HUMAN Q9H3N8 390 aa	[21], potential	20–40	53–73	88–108	132–152	173–193	305–325	342–362
	*H*_5_(*i*), *n* = 5 *u* = 0.25	16–41	55–79	83–107	130–153	169–198	305–331	341–357

RAI3_ HUMAN Q8NFJ5 357 aa	[21], potential	34–54	69–89	98–118	130–150	177–197	213–233	248–268
	*H*_5_(*i*), *n* = 4 〈*u*〉 = 0.195	26–53	68–92	96–118	130–155	178–202	213–233	246–265

VN1R1_ HUMAN Q9GZP7 353 aa	[21], potential	57–77	85–105	133–153	170–190	227–247	275–295	304–324
	*H*_4_(*i*), *n* = 5 〈*u*〉 = 0.754	53–77	90–103	122–145	165–188	222–245	274–301	306–338

APJ_ HUMAN P35414 380 aa	[21], potential	27–51	67–91	101–125	145–166	201–221	245–271	285–308
	*H*_3_(*i*), *n* = 5 *u* = −0.090	30–52	67–85	98–135	147–167	208–228	246– **246–266**	−312 **292–312**

**Table 4:** Prediction of TMD boundaries calculated upon processing of hydrophobicity functions *f*_*n*_(*k*) at *n* = 4, 5 on *H*_*N*_(*i*) (*N* = 3, 4, 5) scales for GPCRs.

Protein name, code, length	Scale level	Number and boundaries of hydrophobic regions, including TMDs						
		1	2	3	4	5	6	7
A4D1U0_ HUMAN A4D1U0 299 aa	*H*_5_(*i*), *n* = 5 〈*u*〉 = 0.439	7–28	45–70	82–102	127–147	173–194	222–240	253–274
	*H*_3_(*i*), *n* = 5 〈*u*〉 = 0.057	7–29	46–71	77–103	124–144	179–194	222–237	258–275

A5Z1T7_ HUMAN A5Z1T7 300 aa	*H*_4_(*i*), *n* = 5 〈*u*〉 = 0.755	7–27	43–57	75–100	121–146	185–210	225–240	263–274
	*H*_3_(*i*), *n* = 5 〈*u*〉 = −0.043	7–26	41–64	75–97	123–144	185–209	225–238	264–274

B5B0C2_ HUMAN B5B0C2 337 aa	*H*_5_(*i*), *n* = 5 〈*u*〉 = 0.142	14–40	49–72	85–122	132–155	189–201	227–255	275–293
	*H*_3_(*i*), *n* = 5 〈*u*〉 = −0.030	14–39	51–71	89– **89–109**	−154 **134–154**	193–205	226–256	277–292

M9TID6_ 9BETA M9TID6 347 aa	*H*_3_(*i*), *n* = 4 *u* = 0.055	43–57	69–88	97–123	149–161	188–219	232–262	265–288
	*H*_5_(*i*), *n* = 5 〈*u*〉 = 0.191	33–56	66–87	100–123	148–164	186–217	233– **233–253**	−295 **275–295**

Q76L88_ HUMAN Q76L88 321 aa	*H*_5_(*i*), *n* = 5 〈*u*〉 = 0.201	11–40	54–78	93–116	156–178	196–223	251–270	
	*H*_3_(*i*), *n* = 5 〈*u*〉 = −0.050	13–37	55–78	90–117	153–174	198–225	248–282	

**Table 5:** Prediction of hydrophobic regions and TMDs calculated upon processing of hydrophobicity functions *f*_*n*_(*k*) at *n* = 4, 5 on *H*_*N*_(*i*) (*N* = 3, 4, 5) scales for α-helical membrane proteins.

Protein name, code, length	Data source	Number and boundaries of hydrophobic regions, including TMDs						
	Scale level	1	2	3	4	5	6	7
SP2Q_ BACSU P71044 283 aa	[21], potential	22–42						
	*H*_5_(*i*), *n* = 5 〈*u*〉 = − 0.127	20–47	70–94	107–124	130–175	207–229		
	*H*_4_(*i*), *n* = 5 〈*u*〉 = 0.696	16–48	70–94	109–121	132–174	197–225		

SP3AH_ BACSU P49785 218 aa	[21], potential	7–26						
	*H*_5_(*i*), *n* = 5 〈*u*〉 = − 0.137	3–31	92–106	146–179	193–211			
	*H*_4_(*i*), *n* = 5 〈*u*〉 = 0.692	3–30	95–113	146–179	193–211			

Q8TMG0_METAC Q8TMG0 194 aa	*H*_5_(*i*), *n* = 5 〈*u*〉 = 0.232	7–20	49–67	76–93	130–162			
	*H*_3_(*i*), *n* = 5 〈*u*〉 = 0.041	0–22	45–62	77–91	127–163			

HLYE_ ECOLI P77335 303 aa	[21], potential					183–203		
	*H*_3_(*i*), *n* = 5 〈*u*〉 = − 0.248	0–17	24–38	82–103	114–123	180–209	242–247	264–280
	*H*_5_(*i*), *n* = 4 〈*u*〉 = 0.029	5–26	32–40	81–102	115–123	179–208	242–253	267–275

**Table 6:** Prediction of TMDs calculated upon processing of hydrophobicity functions *f*_*n*_(*k*) at *n* = 5 on the scale *H*_5_(*i*) for the long α-helical membrane protein.

Protein name, code, length	Data source	Number and boundaries of transmembrane domains					
	Scale	1	2	3	4	5	6
CAC1A_ RABIT P27884 2424 aa	[21], potential	99–117	136–155	168–185	191–209	**229–248**	336–360
	*H*_5_, *u* = 0.305	101–116	141–158	172–185	**210–249**	302–317	336–358
	Scale	7	8	9	10	11	12
	[21], potential	488–506	522–541	550–568	579–597	**617–636**	690–714
	*H*_5_, *u* = 0.305	491–507	518–537	554–577	**609–638**	654–665	685–714
	Scale	13	14	15	16	17	18
	[21], potential	1254–1272	1289–1308	1321–1339	1351–1369	**1389–1408**	1496–1520
	*H*_5_, *u* = 0.305	1255–1270	1293–1312	1323–1339	**1384–1408**	1456–1467	1497–1522
	Scale	19	20	21	22	23	24
	[21], potential	1576–1604	1610–1629	1638–1656	1666–1684	**1704–1723**	1796–1820
	*H*_5_, *u* = 0.305	1575–1599	1607–1633	1641–1660	**1691–1725**	—	1794–1820
