# Corrigendum to “Topography Prediction of Helical Transmembrane Proteins by a New Modification of the Sliding Window Method”

**DOI:** 10.1155/2017/7180293

**Published:** 2017-11-05

**Authors:** Maria N. Simakova, Nikolai N. Simakov

**Affiliations:** ^1^A.F. Mozhaisky Military Space Academy, Yaroslavl 150001, Russia; ^2^Yaroslavl State Technical University, Yaroslavl 150023, Russia

In the article titled “Topography Prediction of Helical Transmembrane Proteins by a New Modification of the Sliding Window Method” [1], the error occurred in formula (1): the summation index *i* of the function *f*_*n*_(*k* + *i*) was accidentally lost. It should be corrected as follows:

(1) fnk=12n+1∑i=−nnfn−1k+i,n=1,2,…,5,f0k=fk.
